# Investigating the Influence of Laser Polarization on Filamentation Thresholds in Transparent Media via Supercontinuum Coherence

**DOI:** 10.3390/s25072285

**Published:** 2025-04-04

**Authors:** Yun Zhang, Yu Xia, Canneng Liang, Yuyao Xiong, Jingyuan Zhang, Shuang Lin, Suyu Li, Mingxing Jin

**Affiliations:** 1 Institute of Atomic and Molecular Physics, Jilin University, Changchun 130012, China; zhangyun18@mails.jlu.edu.cn (Y.Z.); xiayu22@mails.jlu.edu.cn (Y.X.); liangcn22@mails.jlu.edu.cn (C.L.); xiongyy23@mails.jlu.edu.cn (Y.X.); jingyuanz24@mails.jlu.edu.cn (J.Z.); mxjin@jlu.edu.cn (M.J.); 2 Jilin Provincial Key Laboratory of Applied Atomic and Molecular Spectroscopy, Jilin University, Changchun 130012, China; 3 College of Science, Shenyang University of Chemical Technology, Shenyang 110142, China; linshuang20@mails.jlu.edu.cn

**Keywords:** supercontinuum coherence, polarization, femtosecond filamentation, filamentation threshold

## Abstract

In this work, we experimentally investigate the characteristics of supercontinuum (SC) generation induced by femtosecond laser pulses with different polarization states in transparent medium. We employ a Mach–Zehnder Interferometer (MZI) to capture interference patterns during the filamentation process. The relative filamentation threshold, *P*_th_, is measured for femtosecond laser pulses with different polarization states. The results demonstrate that the intensity of SC is strongly correlated with the polarization state of the incident laser pulses. At the same pulse energy, circularly polarized (CP) pulses suppress SC generation compared to linearly polarized (LP) pulses. Compared with weak external focusing, short-focal-length focusing significantly broadens the spectral range of SC. As the focal length of the focusing lens increases, the measured *P*_th_ values also increase. The *P*_th_ of the CP pulses is consistently higher than that of LP pulses. The experimental measurements of *P*_th_ for femtosecond lasers with different polarization states provide basic data support for the research on nonlinear characteristics.

## 1. Introduction

When femtosecond laser pulses propagate in transparent media, the Kerr self-focusing effect occurs when the peak power exceeds the critical power of self-focusing (*P*_cr_). Stable filament structures form when dynamic balance is achieved between Kerr self-focusing and plasma defocusing effects, with the corresponding peak power referred to as the filamentation threshold (*P*_th_). Concurrently, nonlinear effects such as self-phase modulation (SPM) and self-steepening induce significant spectral broadening, generating supercontinuum (SC) radiation spanning from ultraviolet to infrared wavelengths [1,2]. This remarkable broadband characteristic endows SC with significant application potential in diverse fields, including, time-resolved spectroscopy [3], remote sensing [4,5], pulse compression [6], material characterization [7,8], as well as environmental and ecological monitoring [9].

The characteristics of filaments, including SC generation, are highly sensitive to the properties of both the laser source and the propagation medium. Studies demonstrate that parameters such as the initial beam radius [10], pulse duration [11], polarization state of the incident laser [12,13], and external focusing conditions [14] significantly influence filamentation dynamics. Notably, the polarization state of the incident laser affects filament length, diameter, and plasma density. As a key control parameter, the incident laser polarization has been demonstrated to significantly influence the clamped intensity [15], pulse compression [16], plasma formation [17], and SC generation [18,19,20]. Previous research indicates that the fluorescence and luminescence generated during the filamentation process strongly depend on the polarization state of the incident laser pulses [21,22]. For instance, the research group led by Sheng Zhengming [18] investigated the polarization characteristics of SC radiation generated during the long-distance propagation of intense femtosecond laser pulses in air. Their findings reveal that the influence of incident light polarization on SC arises from third-order nonlinear interactions between the laser and the medium. Subsequently, Rostami et al. [23] observed SC generated by elliptically polarized light in a gas medium. They noted that the resulting spectrum exhibited modifications in the SC polarization state and a lengthening of the filament plasma column. In 2020, Wang Tiejun’s research group [24] reported polarization-dependent SC generation in air via filamentation. They proposed a physical picture based on the laser ellipticity-dependent clamped intensity inside the filament and the Kerr nonlinearity and plasma related self-phase modulations to explain their observations.

Recent studies on the influence of incident light polarization on filamentation instabilities reveal a significant increase in the collapse power threshold for circularly polarized (CP) beams [25]. Experimental evidence confirms that using CP light instead of linearly polarized (LP) light can reduce the detrimental impact of the Kerr-nonlinearity, thereby enhancing the self-focusing threshold [26]. Liu et al. [27] demonstrated that the filamentation process is strongly influenced by the polarization state of the driving laser. They experimentally measured the critical power for lasers with different polarization states in air by observing the variation of the laser focal position with the driver laser energy. However, this moving focus method is not applicable to liquid media, limiting its applicability for exploring critical power under diverse polarization states in different media.

Coherence, as a distinctive characteristic of SC radiation, is typically quantified through interferometric measurements of fringe visibility. In 2000, Bellini and Hänsch [28] investigated the coherence of independently generated SC pulse pairs using a modified Young’s double-slit interferometer, obtaining stable and clear interference fringes. In the same year, Dudley and Coen [29] analyzed the fringe visibility at the center of zero-time-delay interference patterns in a similar setup, deriving a mathematical expression for first-order coherence. In 2003, Cook et al. [30] generated one-dimensional arrays of filaments by focusing on water and studied interference effects between adjacent filaments. They observed that the stable interference pattern created by a filament pair is similar to that created by a pair of Young’s slits, and implies a constant phase relationship between the SC generated by each filament. In 2014, Semenova et al. [31] proposed a Michelson interferometer-based method for direct coherence characterization of SC spectra. Based on previous research [32,33], SC can serve as a coherent light source. When it passes through a Mach–Zehnder interferometer (MZI), an interference pattern is formed. This interference pattern provides information related to femtosecond filaments and can be used to determine the onset of filamentation. The MZI technique can be employed to investigate the influence of polarization states on the *P*_th_ in transparent media.

In this study, we investigate the characteristics of the SC induced by femtosecond lasers with different polarization states in transparent media, as well as their filamentation threshold power *P*_th_. In the experiment, by adjusting the laser polarization from linear to circular, we measured the SC induced by the filamentation of laser pulses in liquid media. Subsequently, we collected the interference patterns formed after the SC passed through the MZI, and determined the relative *P*_th_ of femtosecond lasers with different polarization states.

## 2. Experiment Setup

**Figure 1:**
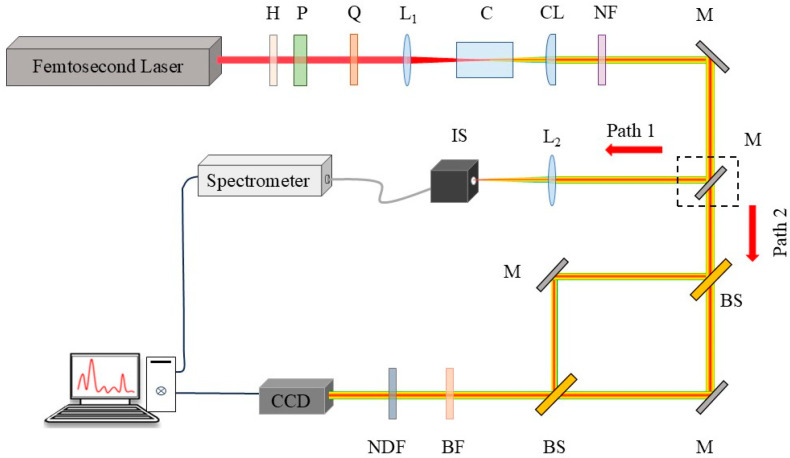
Experimental setup for exploring the influence of polarization on the femtosecond filamentation threshold using a Mach–Zehnder interferometer. H: half-wave plate; P: polarizing beam-splitter; Q: quarter-wave plate; L_1_ and L_2_: focusing lens; C: quartz cuvette with liquid samples; CL: collimating lens (*f* = 75 mm); NF: notch filter; M: plane mirror; IS: integrating sphere; BS: beam splitter; BF: band-pass filter (600 nm); NDF: neutral density filter.

The experimental schematic is depicted in Figure 1. Linearly polarized laser pulses (35 fs, 800 nm, 1 kHz repetition rate) from a Ti: sapphire laser system (Astrella, Coherent Inc., Saxonburg, PA, USA) traverse an energy regulation system composed of a half-wave plate, H, and a polarization beam splitter, P. The incident pulse energy is regulated by rotating the half-wave plate. The incident laser polarization is adjusted by a quarter-wave plate, Q, (QWP) to convert it from linear to elliptical or circular polarization. The input laser beam is focused by lenses, L_1_, with focal lengths of 250 mm, 400 mm, 750 mm, and 1000 mm, respectively. Filaments are generated either in a quartz cuvette, C, (100 mm × 10 mm × 45 mm) filled with liquid or within a solid sample. The SC generated by filamentation is collimated into a parallel beam by a collimating lens, CL. After passing through an 800 nm notch filter, NF, the light beam is directed towards two paths by a movable mirror (the mirror within the black dashed box). Path 1: The SC is converged into the integrating sphere IS by a lens L_2_ (*f* = 100 mm) and then collected by a spectrometer (AvaSpec Fast 1650F-USB2, Avantes BV, Apeldoorn, The Netherlands). Path 2: The beam is split into two paths by a beam splitter, and the optical paths are adjusted to maintain a constant phase difference when the beams are recombined. These devices together constitute the MZI. After the SC passes through the MZI, interference occurs, and the resulting interference fringe pattern is captured by the CCD. A replaceable band-pass filter, BF, (600 nm) and a neutral density filter, NDF, for attenuating the excessive light intensity are placed in front of the CCD.

An energy meter and autocorrelator are used to record the pulse energy and pulse duration when interference patterns are observed. The liquid samples used in the experiment include water, anhydrous ethanol, and a NaCl solution with a concentration of 100 mg/mL. The solid sample is fused silica. The optically transparent samples are selected to prevent the self-absorption of the medium itself from affecting the generated SC. The initial laser is LP, and its polarization direction coincides with the fast axis of the QWP. The angle *θ* between the fast axis and the initial pulse polarization direction can be varied from 0° to 180°. When *θ* = 0° or 90°, the laser pulses after the QWP are LP, and when *θ* = 45°, the laser pulses are CP.

## 3. Results and Discussion

### 3.1. Filament Characteristics

**Figure 2:**
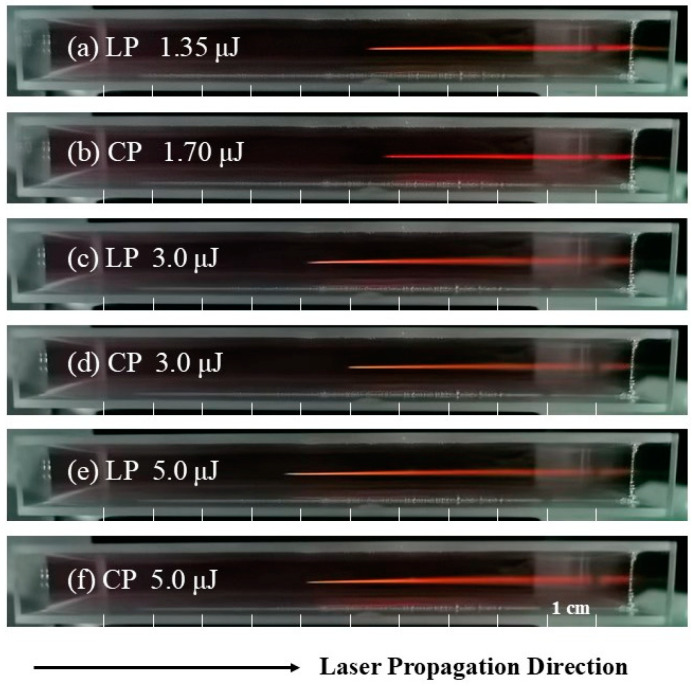
Filaments generated by LP and CP pulses in water (**a**–**f**). The focal length of the lens is 1000 mm, and the cuvette length is 10 cm.

In this experiment, LP and CP pulses are used to generate filaments in the medium. Figure 2 shows the images of filaments generated by LP and CP pulses in water using a focusing lens with *f* = 1000 mm. This focal length was chosen to facilitate the observation of variations in the plasma filament position under different energy conditions. To enhance the visibility of the filamentation process, a small quantity of gold nanoparticles were added to the pure water sample. The filament onset position was set at the center of the cuvette to demonstrate its dependence on both energy and polarization states. However, due to the limited cuvette length, only partial filament development is shown in the Figure 2.

Figure 2a,b show images of the filaments at their initial formation stage induced by LP and CP pulses, respectively. It can be seen that the CP pulse requires more energy to generate a plasma filament as it propagates in water. Moreover, the starting position of the filament generated by the CP pulse is delayed compared to that generated by the LP pulse, which is consistent with the theoretical analyses in references [34,35]. When the initial pulse energy is the same, the filament length generated by the LP pulse is always longer than that of the CP pulse. As the pulse energy gradually increases, the plasma filament moves towards the laser source, as shown in Figure 2c–f. This phenomenon is attributed to the stronger self-focusing effect at higher energies. The third-order nonlinearity effect ${\chi_{\mathrm{cir}}}^{\left(3\right)}$ of CP laser pulses is smaller than the ${\chi_{lin}}^{\left(3\right)}$ of LP laser pulses [15], so the weaker self-focusing ability of CP pulses leads to the delayed filament formation [36,37]. Both the nonlinear refractive index and the multiphoton ionization rate of CP light in the medium are smaller than those of LP light, which results in a shorter filament length and a narrower SC radiation. However, the higher light intensity and plasma density in the filament generated by CP light can partially compensate for the effect of its shorter filament length.

### 3.2. Supercontinuum Generation

In studies on white light generation in isotropic media under laser irradiation, it is prevailing wisdom that the polarization property of white light is consistent with that of incident light [38]. Our objective is to investigate the influence of the laser polarization state on the *P*_th_ through the coherence of SC. We begin by exploring the impact of laser polarization on SC generation.

**Figure 3:**
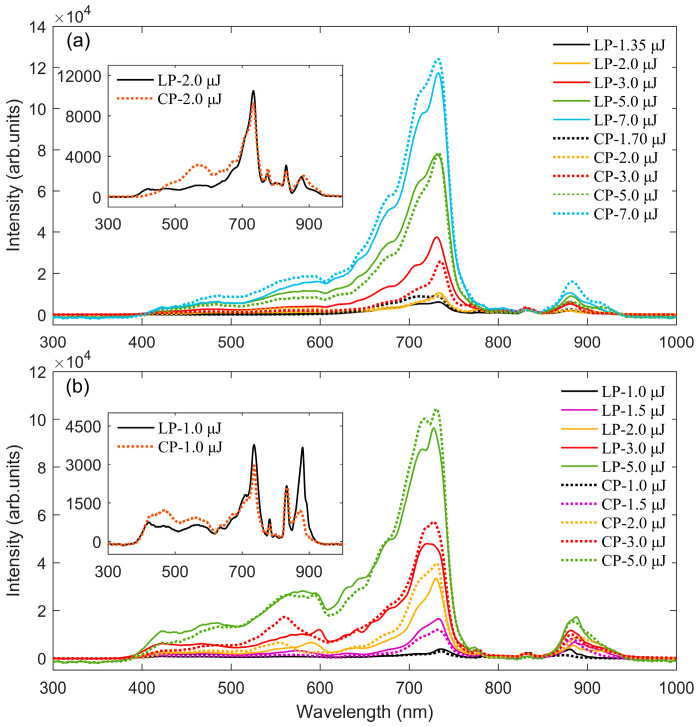
Supercontinuum spectra generated in water by LP and CP incident pulses at different energies. (**a**) SC generated with a focusing lens of *f* = 1000 mm; (**b**) SC generated with a focusing lens of *f* = 250 mm. The inset shows a magnified view of the SC generated at low pulse energy.

The asymmetric broadening on the blue side of the SC can be attributed to self-steepening and plasma-induced self-phase modulation [39]. Figure 3a shows the SC generated under loose focusing with a focal length of *f* = 1000 mm. As the pulse energy increases, the minimum cutoff wavelength $\lambda_{\min}$ of the SC gradually decreases until it reaches a fixed value. This occurs because the spectral broadening range is limited by the clamping intensity within the filament. In loose focusing geometry, the peak intensity within the filament is limited by nonlinear effects [40,41] such as multiphoton absorption and plasma defocusing. Figure 3b illustrates the SC generated under short-focal-length focusing (*f* = 250 mm). Compared with loose focusing, the $\lambda_{\min}$ of the spectra generated under short-focal-length focusing exhibit a blue shift. In short-focal-length focusing geometry, the high initial beam curvature caused by strong external focusing leads to the complete ionization of the medium. This prevents plasma defocusing from playing an important role in intensity clamping [42], and the peak intensity within the filament greatly exceeds the clamping intensity. Therefore, compared with loose focusing, the short-focal-length focusing significantly broadens the spectral range of the SC.

For LP and CP pulses with the same energy, the overall spectral content and spectral broadening of the SC are nearly identical. However, the intensity of the generated wavelengths is dependent on the polarization state of the laser pulses. As shown in the inset of Figure 3a, when the same pulse energy as the LP pulse is applied, the CP pulse suppresses SC generation. This is consistent with the findings of Sandhu et al. [19] The suppression of SC by CP pulses can be attributed to the weaker third-order nonlinear effect of CP pulses in the medium, as well as other nonlinear optical processes that are more sensitive to the polarization state. Compared with CP pulses, medium ionization induced by LP pulses with the same energy has a higher rate, resulting in higher plasma enhanced self-phase modulation and a more pronounced blue shift [18].

Taking the case of *f* = 250 mm as an example, as shown in Figure 3b, at low pulse energy (e.g., 1.0 μJ, 1.5 μJ), the SC signal intensity generated by the LP pulse is higher than that generated by the CP pulse. When the laser peak power significantly exceeds the critical power for filamentation, the SC signal intensity generated by CP pulse is higher, consistent with the findings of Yang et al. [18] The SC signal intensity is directly related to the intensity inside the laser filament. Simulations by Panov and co-workers [13] have demonstrated that the clamping intensity under circular polarization is higher than that under linear polarization. As the polarization state of the incident laser changes from linear to circular, the higher ionization ratio and critical power for self-focusing [43,44] result in more laser energy being confined within the filament. The polarization state of the incident laser pulses directly affects the clamping intensity during the filamentation process. Therefore, compared with the LP pulses, the clamping intensity of CP pulses during the filamentation process is enhanced, resulting in a higher signal intensity of the SC. The same phenomenon is also observed under the condition of *f* = 1000 mm (see Figure 3a). However, under this long-focal-length condition, the laser energy required for signal intensity inversion is higher. This is because, as the focal length increases, the energy required for laser pulses to form filaments in the medium also rises accordingly. The signal intensity inversion is not the primary focus of this study, its underlying mechanisms will be explored in future work.

**Figure 4:**
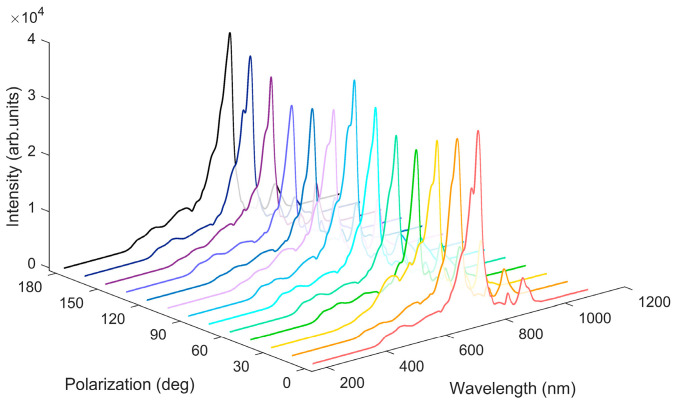
SC generated by femtosecond laser pulses with different polarization states in water. The pulse energy is 3.0 μJ, and a focusing lens of *f* = 1000 mm is used.

The SC generated by femtosecond laser pulses with different polarization states in water is shown in Figure 4. The laser pulse energy is fixed at 3.0 μJ. As the rotation angle of the QWP changes, the intensity of the SC generated in water oscillates periodically at an interval of 90°. The maximum signal intensity of the SC occurs at *θ* = 0°, 90°, and 180°, corresponding to the LP pulses; the minimum occurs at *θ* = 45° and 135°, corresponding to the CP pulses. The signal intensity of the SC depends on the polarization of the incident laser pulses and increases as the polarization state changes from circular to linear, independent of the sample [20].

### 3.3. Filamentation Threshold

**Figure 5:**
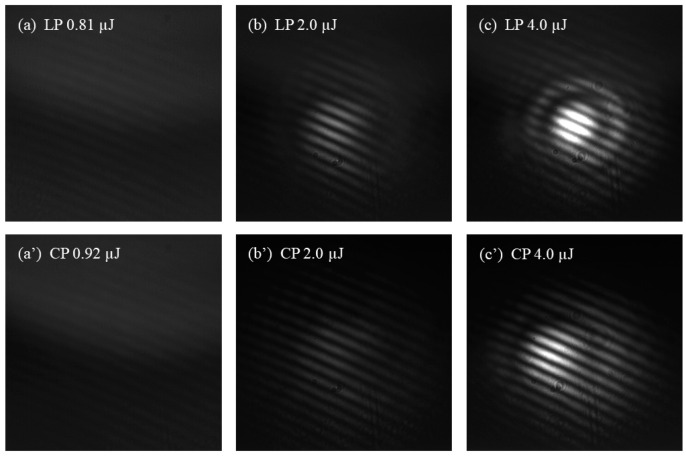
Interference patterns generated by LP and CP pulses in water. A focusing lens of *f* = 400 mm is used.

Femtosecond filamentation is accompanied by the generation of SC. In our previous research [45], we discussed the drastic changes in the spectral range of the SC near the filamentation threshold (*P*_th_). The onset of femtosecond filamentation can be characterized by the appearance of SC. However, due to the limited detection efficiency of the spectrometer, some spectral components are undetectable at low pulse energies. Therefore, determining the *P*_th_ solely by spectroscopic technology is insufficiently accurate. The SC can serve as a coherent light source. When the SC passes through the MZI, equal-thickness interference occurs, forming an interference pattern. The interference pattern provides information about femtosecond filaments and can be used to determine the onset of filamentation. Considering that the diffraction effect may distort and even mask the interference pattern, we used a 600 nm band-pass filter in the experiment. The spectral signals of shorter wavelengths can eliminate the influence of diffraction [45]. In the experiment, interference patterns resulting from the SC generated by LP and CP pulses in the medium are captured by a CCD. The energy at the appearance of the interference patterns is measured by a pulse energy meter, and this pulse power is defined as the *P*_th_. Figure 5 illustrates the interference patterns generated by LP and CP laser pulses in water after passing through a lens with a focal length of 400 mm.

**Figure 6:**
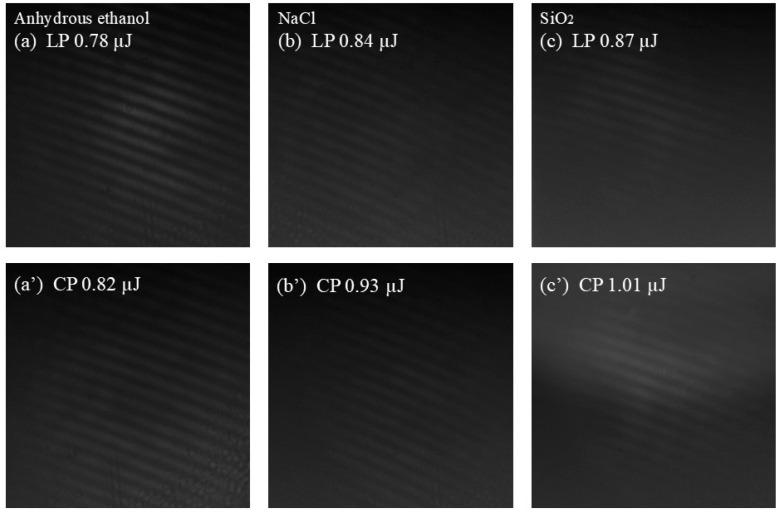
Interference patterns generated by LP and CP pulses in anhydrous ethanol, NaCl solution, and SiO₂. A focusing lens of *f* = 400 mm is used.

The energy of the incident laser pulse is adjusted by rotating the half-wave plate. When the pulse power is below the *P*_th_, the SC cannot be generated, and no image is visible in the field of view. As the pulse energy increases, flickering interference fringes begin to appear. The appearance of these interference fringes indicates filament formation in the water and the generation of SC. When the input pulse is LP, the energy required for the interference fringes to become visible in the field of view is approximately 0.81 µJ, as shown in Figure 5a. For CP laser pulses, the energy required for filamentation is approximately 0.92 µJ, as shown in Figure 5a’. As the pulse energy increases to 2.0 µJ, the interference fringes become clearer and more stable. However, further increasing the pulse energy causes the interference patterns to distort. This distortion is likely caused by the formation of multiple filaments and their mutual interference, as shown in Figure 5b,c. Furthermore, we captured the interference patterns generated during the filamentation of LP and CP pulses in anhydrous ethanol, NaCl solution with a concentration of 100 mg/mL, and fused silica, as depicted in Figure 6. The results indicate that the energy required for filamentation with CP pulses is greater than that for LP pulses, meaning the *P*_th_ for CP pulses is higher than that for LP pulses.

**Figure 7:**
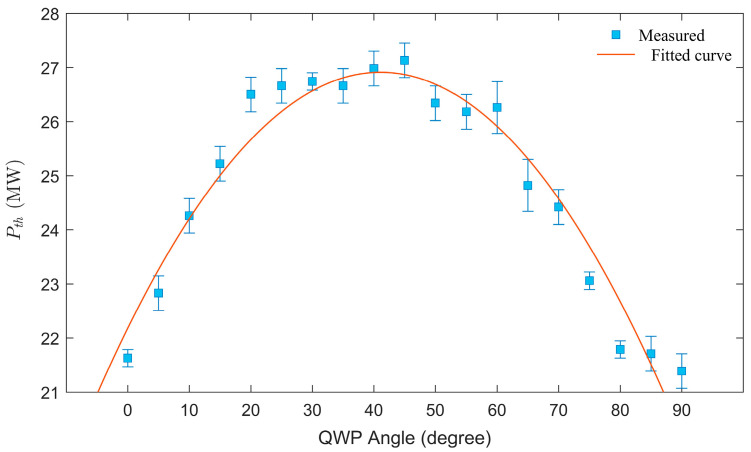
Variation of the measured *P*_th_ in water with laser polarization state. The horizontal axis represents the rotation angle of the QWP. A focusing lens of *f* = 1000 mm is used.

We further investigate the *P*_th_ of laser pulses in water under various polarization states, as shown in Figure 7. The horizontal axis represents the rotation angle of the QWP, corresponding to different polarization states: 0° and 90° indicate linear polarization, 45° denotes circular polarization, and other angles represent elliptical polarization with varying ellipticity. The data reveal that the *P*_th_ for elliptically polarized pulses lies between that of linear and circular polarization, and the threshold for CP pulses is significantly higher than that for LP pulses. The experimental data in Figure 7 are fitted with a sine function, and the *P*_th_ is approximately 21.63 MW for LP pulses and 27.13 MW for CP pulses. The measured *P*_th_ for LP pulses is quite close to the previously reported experimental value of 22.02 MW [32]. The variation trend of the *P*_th_ in water with respect to laser polarization is similar to the research on the critical power of self-focusing in air by Liu et al. [27] The *P*_th_ of CP pulses is higher than that of LP pulses, due to the smaller nonlinear refractive index of the medium for CP incident lasers.

The self-focusing collapse propagation distance, *L*_c_, of a Gaussian beam can be well approximated by the semiempirical formula [46,47]: $L_{c}=\frac{0.367ka_{0}^{2}}{\sqrt{{\left[{\left(P_{\mathrm{in}}/P_{\mathrm{cr}}\right)}^{1/2}-0.852\right]}^{2}-0.0219}}$. For a beam focused by a lens with a focal length of *f*, the collapse distance can be estimated by the formula [48]: $L_{cf}=\frac{L_{c}\cdot f}{L_{c}+f}$. That is to say, when the initial pulse energy is the same, the collapse distance increases with the *P*_cr_. In other words, as the initial pulse polarization changes from linear to elliptical and then to circular, the critical power of self-focusing gradually increases, and the starting position of the filament moves further away from the focusing lens. This behavior is consistent with the experimental results shown in Figure 2.

**Figure 8:**
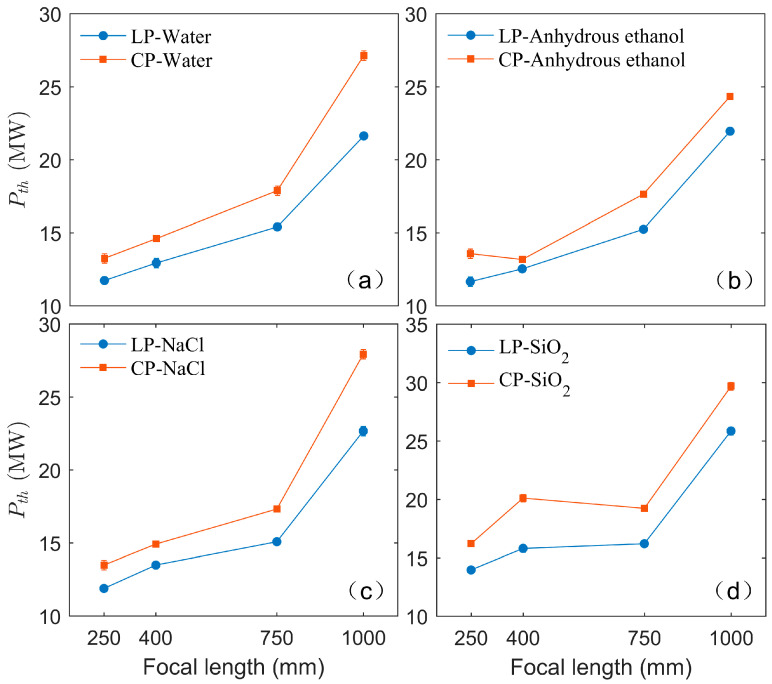
Variations of the filamentation thresholds of LP and CP pulses with focal length in (**a**) water, (**b**) anhydrous ethanol, (**c**) NaCl solution, and (**d**) fused SiO₂.

In this study, the onset of filamentation is determined by observing the emergence of interference patterns, and the corresponding pulse energy is measured using a pulse energy meter. The femtosecond, *P*_th_, can be calculated according to the formula: $P=E_{in}/(\sqrt{\pi /2}\tau_{p})$, where $\tau_{p}$ is the pulse duration. The initial pulse duration is 35 fs. After filamentation in the medium and passing through the interference system, the duration of the original CP/LP pulses broaden. The apparent broadening of the pulse duration after filamentation can be attributed to angular dispersion and temporal dispersion caused by filamentation [49]. The broadened pulse duration is measured to be approximately 50 fs using an autocorrelator. It should be noted that the measured pulse energy exhibits slight fluctuations, likely due to fluctuations in the incident femtosecond laser pulse energy and the limited detection precision of the energy meter. These fluctuations in pulse energy result in a corresponding variability in the measured *P*_th_, as indicated by error bars in Figure 7. Table 1 shows the measured *P*_th_ values for filamentation of LP and CP pulses in four media using focusing lenses with different focal lengths. The relationship between the *P*_th_ and lens focal length is illustrated in Figure 8.

Figure 8 presents the variations of the *P*_th_ of LP and CP pulses with focal length. It is evident that the *P*_th_ for both LP and CP pulses increases with focal length. This phenomenon may be attributed to the effect of focal length on filamentation dynamics. For a long focal length (*f* = 1000 mm), the converging ability of the lens reduces, and the self-focusing effect plays a more dominant role during the filamentation process. As a result, more energy is required for filament formation. For a short focal length, the beam convergence is strongly influenced by the lens, and the filamentation process is primarily determined by linear focusing. Consequently, the energy required for filament formation is lower than that for long focal lengths. Furthermore, regardless of focal length changes, the *P*_th(cir)_ of the CP pulses consistently exceeds the *P*_th(lin)_ of the LP pulses. In comparison to LP pulses, both the self-focusing effect and the nonlinear absorption effect for CP pulses are relatively weaker.

A focusing lens with a focal length of 100 mm is also used in the experiment. However, the difference between the measured *P*_th_ of LP and CP pulses is extremely small, so the data are not presented in this paper. When the focal length is relatively small, the filamentation process exhibits a characteristic of being insensitive to the polarization state of the laser pulses. Under short-focal-length focusing conditions, the shortened interaction distance causes linear focusing to dominate over nonlinear self-focusing, resulting in negligible differences in the axial intensity distribution between the LP and CP cases. Less electron plasma is generated during linear transmission, leading to reduced energy absorption and scattering at the trailing edge of the laser pulses. This results in a higher axial intensity compared to the nonlinear case. Compared with using a lens with *f* = 250 mm, the measured pulse duration is wider when using a lens with *f* = 1000 mm. This may be due to the fact that a significant self-phase modulation effect generates more positive chirp, while the plasma effect produces less negative chirp [50]. Therefore, the lens with *f* = 1000 mm introduces an additional positive chirp to the filamentation process, which may be the reason for the pulse broadening. That is to say, for long focal lengths, if the influence of the lens focal length on pulse broadening is strictly considered and a larger pulse duration value is used in calculating the threshold, the obtained *P*_th_ would be slightly smaller than the values in Table 1. However, this does not alter the overall trend. The purpose of this study is to provide an effective method for investigating the influence of the polarization state on the critical power of filamentation. Although the research process may be limited by the detection efficiency of the CCD, the relative measured values of *P*_th_ obtained by this method remain valuable for reference.

## 4. Conclusions

In this study, a MZI detection system is employed to collect SC and interference patterns generated by laser pulses with different polarization states in transparent media, and the relative *P*_th_ is measured. The influence of different focusing conditions on the polarization-dependent SC characteristics and the measured threshold is also investigated. The experimental findings are as follows: the intensity of the SC signal is strongly correlated with the polarization state of the incident laser pulses. Under identical energy conditions, CP pulses suppress SC generation compared to LP pulses. This polarization dependence of SC is universal and independent of the sample selection. Furthermore, variations in the focal length of the focusing lens influence filamentation dynamics, resulting in changes in the measured *P*_th_. Meanwhile, short-focal-length focusing conditions demonstrate a more significant enhancement effect on the SC spectral range compared to weak external focusing. Through experimental measurement and fitting analysis of the *P*_th_ for different polarization states, it is observed that the *P*_th_ of CP pulses is consistently higher than that of LP pulses. The experimental measurement of the *P*_th_ of femtosecond lasers with different polarization states provides basic data support for the research on nonlinear optical characteristics. Subsequently, we intend to use the interferometry method to explore more complex physical processes to expand its practical applications.

## Figures and Tables

**Table 1:** Filamentation thresholds *P*_th_ of LP and CP pulses in four media when using focusing lenses with different focal lengths.

Material	Polarization	*P*_th_ (MW)			
		*f* = 250 mm	*f* = 400 mm	*f* = 750 mm	*f* = 1000 mm
Water	LP	11.73	12.93	15.40	21.63
	CP	13.25	14.60	17.88	27.13
Anhydrous ethanol	LP	11.65	12.53	15.24	21.95
	CP	13.57	13.17	17.64	24.34
NaCl (100 mg/mL)	LP	11.89	13.49	15.08	22.67
	CP	13.49	14.92	17.32	27.93
SiO_2_	LP	13.97	15.80	16.20	25.86
	CP	16.20	20.11	19.23	29.69

## Data Availability

The data presented in this study are available on reasonable request from the corresponding author.

## References

[1] Xu H.L., Chin S.L. (2011). Femtosecond laser filamentation for atmospheric sensing. Sensors.

[2] Yang L., Zhang B., Wu T., Zhao Y., Hou J. (2018). Watt-level mid-infrared supercontinuum generation from 2.7 to 4.25 μm in an erbium-doped ZBLAN fiber with high slope efficiency. Opt. Lett..

[3] Swartling J., Bassi A., D’Andrea C., Pifferi A., Torricelli A., Cubeddu R. (2005). Dynamic time-resolved diffuse spectroscopy based on supercontinuum light pulses. Appl. Opt..

[4] Rairoux P., Schillinger H., Niedermeier S., Rodriguez M., Ronneberger F., Sauerbrey R., Stein B., Waite D., Wedekind C., Wille H. (2000). Remote sensing of the atmosphere using ultrashort laser pulses. Appl. Phys. B.

[5] Kasparian J., Sauerbrey R., Mondelain D., Niedermeier S., Yu J., Wolf J.P., André Y.B., Franco M., Prade B., Tzortzakis S. (2000). Infrared extension of the super continuum generated by femtosecond terawatt laser pulses propagating in the atmosphere. Opt. Lett..

[6] Cheng C., Zeng Y., Ou Y., Lv H., Lv Q., Zhang J. (2017). Few-cycle pulse generation from compression of supercontinuum by optimizing initial chirp in ANDi-PCF. Optik.

[7] De Boni L., Andrade A., Misoguti L., Mendonça C., Zilio S. (2004). Z-scan measurements using femtosecond continuum generation. Opt. Express.

[8] Balu M., Hales J., Hagan D.J., Stryland E.V. (2004). White-light continuum Z-scan technique for nonlinear materials characterization. Opt. Express.

[9] Kasparian J., Rodriguez M., Méjean G., Yu J., Salmon E., Wille H., Bourayou R., Frey S., André Y.B., Mysyrowicz A. (2003). White-light filaments for atmospheric analysis. Science.

[10] Luo Q., Hosseini S.A., Liu W., Gravel J.F., Kosareva O.G., Panov N.A., Aközbek N., Kandidov V.P., Roy G., Chin S.L. (2005). Effect of beam diameter on the propagation of intense femtosecond laser pulses. Appl. Phys. B.

[11] Liu W., Chin S.L. (2005). Direct measurement of the critical power of femtosecond Ti: Sapphire laser pulse in air. Opt. Express.

[12] Petit S., Talebpour A., Proulx A., Chin S.L. (2000). Polarization dependence of the propagation of intense laser pulses in air. Opt. Commun..

[13] Panov N.A., Kosyreva O.G., Savel’ev A.B., Uryupina D.S., Perezhogin I.A., Makarov V.A. (2011). Filamentation of femtosecond Gaussian pulses with close-to-linear or-circular elliptical polarization. Quantum Electron..

[14] Théberge F., Liu W., Simard P.T., Becker A., Chin S.L. (2006). Plasma density inside a femtosecond laser filament in air: Strong dependence on external focusing. Phys. Rev. E.

[15] Vuong L.T., Grow T.D., Ishaaya A., Gaeta A.L., ’tHooft G.W., Eliel E.R., Fibich G. (2006). Collapse of optical vortices. Phys. Rev. Lett..

[16] Varela O., Zaïr A., Román J.S., Alonso B., Sola I.J., Prieto C., Roso L. (2009). Above-millijoule super-continuum generation using polarisation dependent filamentation in atoms and molecules. Opt. Express.

[17] Kolesik M., Moloney J.V., Wright E.M. (2001). Polarization dynamics of femtosecond pulses propagating in air. Phys. Rev. E.

[18] Yang H., Zhang J., Zhang Q., Hao Z., Li Y., Zheng Z., Wang Z., Dong Q., Lu X., Wei Z. (2005). Polarization-dependent supercontinuum generation from light filaments in air. Opt. Lett..

[19] Sandhu A.S., Banerjee S., Goswami D. (2000). Suppression of supercontinuum generation with circularly polarized light. Opt. Commun..

[20] Srivastava A., Goswami D. (2003). Control of supercontinuum generation with polarization of incident laser pulses. Appl. Phys. B.

[21] Zhang H., Jing C., Li G., Xie H., Yao J., Zeng B., Chu W., Ni J., Xu H., Cheng Y. (2013). Abnormal dependence of strong-field-ionization-induced nitrogen lasing on polarization ellipticity of the driving field. Phys. Rev. A.

[22] Mitryukovskiy S., Liu Y., Ding P., Houard A., Couairon A., Mysyrowicz A. (2015). Plasma luminescence from femtosecond filaments in air: Evidence for impact excitation with circularly polarized light pulses. Phys. Rev. Lett..

[23] Rostami S., Chini M., Lim K., Palastro J.P., Durand M., Diels J.C., Arissian L., Baudelet M., Richardson M. (2016). Dramatic enhancement of supercontinuum generation in elliptically-polarized laser filaments. Sci. Rep..

[24] Chen N., Wang T.J., Zhu Z., Guo H., Liu Y., Yin F., Sun H., Leng Y., Li R. (2020). Laser ellipticity-dependent supercontinuum generation by femtosecond laser filamentation in air. Opt. Lett..

[25] Bergé L., Gouédard C., Schjødt-Eriksen J., Ward H. (2003). Filamentation patterns in Kerr media vs. beam shape robustness, nonlinear saturation and polarization states. Phys. D.

[26] Schimpf D.N., Eidam T., Seise E., Hädrich S., Limpert J., Tünnermann A. (2009). Circular versus linear polarization in laser-amplifiers with kerr-nonlinearity. Opt. Express.

[27] Liu C., Zang H., Li H., Yu Y., Xu H. (2017). Polarization effect on critical power and luminescence in an air filament. Chin. Opt. Lett..

[28] Bellini M., Hänsch T.W. (2000). Phase-locked white-light continuum pulses: Toward a universal optical frequency-comb synthesizer. Opt. Lett..

[29] Dudley J.M., Coen S. (2002). Coherence properties of supercontinuum spectra generated in photonic crystal and tapered optical fibers. Opt. Lett..

[30] Cook K., Kar A.K., Lamb R.A. (2003). White-light supercontinuum interference of self-focused filaments in water. Appl. Phys. Lett..

[31] Semenova V.A., Tsypkin A.V., Putilin S.E., Bespalov V.G. (2014). A method for the coherence measurement of the supercontinuum source using Michelson interferometer. J. Phys. Conf. Ser..

[32] Zhang Y., Xia Y., Liang C., Chen A., Li S., Jin M. (2023). Exploring the femtosecond filamentation threshold in liquid media using a Mach–Zehnder interferometer. Sensors.

[33] Zhang H., Zhang Y., Lin S., Chang M., Yu M., Wang Y., Chen A., Jiang Y., Li S., Jin M. (2021). Testing the coherence of supercontinuum generated by optical vortex beam in water. J. Phys. B At. Mol. Opt. Phys..

[34] Fibich G., Ilan B. (2000). Self-focusing of elliptic beams: An example of the failure of the aberrationless approximation. J. Opt. Soc. Am. B.

[35] Kandidov V.P., Fedorov V.Y. (2004). Properties of self-focusing of elliptic beams. Quantum Electron..

[36] Nuter R., Skupin S., Bergé L. (2005). Chirp-induced dynamics of femtosecond filaments in air. Opt. Lett..

[37] Sutherland R.L. (1996). Handbook of Nonlinear Optics.

[38] Alfano R.R., Shapiro S.L. (1970). Observation of self-phase modulation and small-scale filaments in crystals and glasses. Phys. Rev. Lett..

[39] Gaeta A.L. (2000). Catastrophic collapse of ultrashort pulses. Phys. Rev. Lett..

[40] Liu W., Petit S., Becker A., Aközbek N., Bowden C.M., Chin S.L. (2002). Intensity clamping of a femtosecond laser pulse in condensed matter. Opt. Commun..

[41] Couairon A., Chakraborty H.S., Gaarde M.B. (2008). From single-cycle self-compressed filaments to isolated attosecond pulses in noble gases. Phys. Rev. A.

[42] Kiran P.P., Bagchi S., Arnold C.L., Krishnan S.R., Kumar G.R., Couairon A. (2010). Filamentation without intensity clamping. Opt. Express.

[43] Panov N.A., Makarov V.A., Fedorov V.Y., Kosareva O.G. (2013). Filamentation of arbitrary polarized femtosecond laser pulses in case of high-order Kerr effect. Opt. Lett..

[44] Zhang X., Wang T.J., Guo H., Chen N., Lin L., Zhang L., Sun H., Liu J., Liu J., Shen B. (2019). Polarization sensitive laser intensity inside femtosecond filament in air. arXiv.

[45] Li S., Wang X., Zhang Y., Yu M., Wang Y., Liu F., Jin M. (2023). Femtosecond filamentation in water studied by the interference of supercontinuum. Phys. Scr..

[46] Couairon A., Mysyrowicz A. (2007). Femtosecond filamentation in transparent media. Phys. Rep..

[47] Marburger J.H. (1975). Self-focusing: Theory. Prog. Quantum Electron..

[48] Talanov V.I. (1970). Focusing of Light in Cubic Media. JETP Lett..

[49] Gu X., Akturk S., Trebino R. (2004). Spatial chirp in ultrafast optics. Opt. Commun..

[50] Guo H., Dong X., Wang T.J., Zhang X., Chen N., Yin F., Wang Y., Zhang L., Sun H., Liu J. (2021). Polarization dependent clamping intensity inside a femtosecond filament in air. Chin. Opt. Lett..

